# Optical assessment of lignin-containing nanocellulose films under extended sunlight exposure

**DOI:** 10.1007/s10570-025-06380-7

**Published:** 2025-01-15

**Authors:** Rustem Nizamov, Joice Kaschuk, Yazan Al Haj, Mikael Nyberg, Monireh Imani, Eva Pasquier, Orlando Rojas, Tiffany Abitbol, Jaana Vapaavuori, Kati Miettunen

**Affiliations:** 1 Department of Mechanical and Materials Engineering, Faculty of Technology, University of Turku, 20500 Turku, Finland; 2 Department of Bioproducts and Biosystems, School of Chemical Engineering, Aalto University, Vuorimiehentie 1, 02150 Espoo, Finland; 3 Department of Chemical and Biological Engineering, The University of British Columbia, 2360 E Mall, Vancouver - BC, V6T 1Z3 Canada; 4 Physical Chemistry and Soft Matter, Wageningen University and Research, 6708 WE Wageningen, The Netherlands; 5 Department of Chemistry and Materials Science, School of Chemical Engineering, Aalto University, Kemistintie 1, 02150 Espoo, Finland; 6 Mirka Ltd, Pensalavägen 210, FI-66850 Jeppo, Finland; 7 Institute of Materials, School of Engineering, École Polytechnique Fédérale de Lausanne (EPFL), 1015 Lausanne, Switzerland

**Keywords:** Stability, Optoelectronics, Mechanical properties, UV protection, Biobased solar cells

## Abstract

This study investigates the stability and UV-blocking properties of cellulose nanofibril (CNF) and TEMPO-oxidized cellulose nanofibril (TOCNF) films, with and without lignin, under 1000 h of artificial sunlight. The literature to date provides no quantitative analysis of such films’ stability, however such insight is critical for optoelectronic applications for instance solar cells. This contribution examines the films from practical perspectives, considering aging with respect to their optical performance and retention of UV protective qualities. Films containing residual lignin (LignoCNF and LignoTOCNF), and lignin nanoparticles (CNF-LNP and TOCNF-LNP) demonstrated remarkable UV-blocking stability; even after the aging transmittance of LignoCNF and CNF-LNP films remained lower than 1% below 390 nm. Most lignin-containing films exhibited increased transmittance between 400 and 600 nm after aging, except for LignoTOCNF, which showed a decrease in transmittance that was comparable to that displayed by non-lignin films. Nevertheless, long-term light exposure induced a decrease in their mechanical properties. Tensile tests revealed increased brittleness in CNF and LignoCNF, while LNP-containing films showed reduced strain at the break. The observed changes were linked to the potential oxidation of COO- groups and structural modifications in both cellulose and lignin. Overall, the incorporation of lignin into nanocellulose films enhances their durability, UV protection, and mechanical stability, making them promising candidates for sustainable optoelectronic applications.

**Supplementary Information:**

The online version contains supplementary material available at 10.1007/s10570-025-06380-7.

## Introduction

Nanocellulose (NC) films have been considered as potentially applicable for electronic applications due to their abundance, and bendability, among other factors (Suresh Khurd and Kandasubramanian 2022). NC produces highly transparent and dense films that have the potential to support light management in optoelectronics (Tarrés et al. 2019; Kaschuk et al. 2022; Banvillet et al. 2023). Its structure can be adjusted by modulating 3D-nanoporosity and surface topology, achieving broadband anti-reflection, diffuse light transmission (haze) and controlled structural colors (Moon et al. 2022). Recent studies have investigated, in particular, the impact of haze and the engineering of refractive indexes to improve light harvesting and, thereby, solar cell performance (Wu et al. 2020; Hou et al. 2020; Miettunen et al. 2024; Kaschuk et al. 2024). Generally, high haze does not inherently improve optical performance, but combined with modifications to microstructure and/or porosity, it could lead to an engineered refractive index, altering scattering direction and enhancing material performance (Kaschuk et al. 2024).

However, the advantages of using NC films for optoelectronics go beyond their optical properties. For instance, glass, used to produce solar cells modules, is submitted to extreme temperatures (1200–1600 °C) contributing to high CO_2_ emissions (around 86Mt) but at low costs ($7/m^2^) (Horowitz et al. 2016; Westbroek et al. 2021). Already, NC can be produced at lower temperature inducing to low energetic costs (< $10/kg) and lower CO_2_ emissions (1.8–1100 kg CO_2_-eq/kg) (Serra et al. 2017; Kane et al. 2023). Furthermore, the straightforward processability and scalability of NC films, facilitated by papermaking methodology, allow efficient large-scale production of flexible optoelectronics (Li et al. 2021). Finally, considering the device’s life cycle, the replacement of conventional optoelectronic glass substrates with biobased materials can support the retrieval of rare and expensive elements from these devices at the end of life (Miettunen and Santasalo-Aarnio 2021; Akulenko et al. 2023; Miettunen et al. 2024).

Overall, chemically modified cellulose nanofibril (CNF) such as TEMPO-oxidized cellulose nanofibril (TOCNF) are usually more transparent and have different haze characteristics when compared to films from unmodified CNF (Kaschuk et al. 2022). TEMPO oxidation, a regioselective modification that converts C6 hydroxyl groups of cellulose into carboxyl groups (Saito et al. 2007), produces nanofibrils with high aspect ratios (> 50) and excellent mechanical properties (tensile strength: 200–300 MPa) advantageous for optoelectronic applications (Tang et al. 2024).

Furthermore, light management properties, such as UV protection, can be incorporated to NC films which helps to prevent degradation of the active layer in optoelectronic devices, ultimately extending their operational lifespan while maintaining efficiency (Poskela et al. 2021). The UV shielding property is achieved by incorporating semiconductor nanoparticles (Abitbol et al. 2020) or biobased particles, such as lignin (Pasquier et al. 2021). Macromolecular lignin contains phenolic units, ketones, and other chromophores that are responsible for absorbing UV light (Kaschuk et al. 2022). In nature, lignin is constantly renewed by means of complex biosynthesis, and thus the UV protection of plants remains stable over lengthy periods of time (Cogulet et al. 2016). Therefore, this study also asks whether lignin, after being removed from the plants and processed into films, retains long-lasting UV protection. To address this, we used NC previously produced and studied containing residual lignin (Imani et al. 2019) as well as NC films where lignin nanoparticles were nucleated onto the fibers surface (Pasquier et al. 2021).

Altogether, this study provides valuable insights into the long-term optical (including UV shielding property) and mechanical stability of NC films. These insights are not presented in the literature as highlighted by Pan et al. (Pan et al. 2023), and they are crucial to ensuring the performance of practical commercial applications. By exploring fundamental aspects of materials science alongside practical light exposure testing relevant for photovoltaics, this research offers new valuable insights into the suitability of NC films for optoelectronics applications as well as their behaviour under exposure to light.

## Materials and methods

### NC description

All NC were produced at the Department of Bioproducts and Biosystems, Aalto University using a high-pressure fluidizer (pressure of 1500 bars, Microfluidics M110P, Microfluidics Int. Co., Newton, MA). Both CNF and TOCNF were produced from never dried, fully bleached, and fines-free sulphite birch pulp (Kappa number = 1, and DP = 4700). CNF was disintegrated six passes, and TOCNF was produced by TEMPO-mediated oxidation (2,2,6,6-tetramethylpiperidine-1-oxyl) (Saito et al. 2007) and one pass fibrillation. For the residual containing lignin NC samples, an unbleached softwood (20% recycled fibers) (1/3 Spruce and 2/3 Pine) Kraft pulp was used (supplied by Stora Enso, Finland) following similar methodology described for TOCNF and CNF. Technical kraft lignin (Indulin AT) from softwood was obtained from MeadWestvaco.

### Films preparation

Six 60 g·m^−2^ NC films were produced, divided into two groups: cellulose nanofibrils (CNF) and tempo-oxidized cellulose nanofibrils (TOCNF). Each group had three films: one without lignin, one with added lignin nanoparticles (LNPs), and one with residual lignin (Ligno). The films were produced by air pressure filtration followed by pressing (30 °C) following the procedure used in our previous work (Kaschuk et al. 2024). To produce NC-LNP films, the lignin was dissolved and dropped into the NC suspension. This caused the nucleation of the lignin on the surface of NC fibers which is described on (Pasquier et al. 2021).

Both the Ligno-CNF and Ligno-TOCNF samples had a lignin content between 13 and 15%, while both CNF-LNP and TOCNF-LNP presented a final lignin content of approximately 9%. More information can be found in our previous works (Imani et al. 2019; Pasquier et al. 2021).

### Optical microscopy

Structural morphology assessment of NC films was performed using an upright optical microscope (bScope materials science, Euromex). The microscope is equipped with wide-field WF 10x/22 mm eyepieces and a Plan PLMi 10x/0.25 infinity-corrected IOS objective (BS.8110) with a working distance of 20.2 mm. The NC film samples were cut into small rectangular pieces and placed between two rectangular glass slides, forming a sandwich structure to ensure uniform flatness during imaging.

### Field-emission scanning electron microscopy

Surface topography and structural details of the NC films were examined using a field-emission scanning electron microscope (FESEM, Apreo S, Thermo Scientific). The samples were cut into small pieces and mounted on aluminum stubs using copper tape and conductive carbon paste. Since the samples are inherently non-conductive, a 6 nm layer of platinum was deposited onto the sample surfaces using a Quorum Q150V ES + sputter coater to provide the necessary conductivity for imaging. Imaging was conducted using a beam acceleration voltage of 2 kV, with the Everhart–Thornley detector capturing secondary electron images. The full horizontal field width of the images was set to approximately 30 µm.

### Artificial sunlight exposure

The films were aged in a solar simulator chamber (Suntest XLS + , by Atlas) with a xenon lamp (model NXE 1700) simulating the AM 1.5G solar spectrum (Abitbol et al. 2023). The weather chamber is equipped with cooling fans to cool down the xenon lamp. During the experiment, a relative humidity of approximately 10% was measured inside the chamber. The films were exposed to artificial sunlight for 1000 h at an average temperature of 45 °C. This duration was selected as it reflects typical light soaking tests in photovoltaics, representing approximately one year of exposure in central European outdoor conditions (Osterwald and McMahon 2009).

### Color alteration assessment

The methodology used to capture and analyze the color alterations in the films follows the procedures described by Lawrynowicz et al. (Lawrynowicz et al. 2024). Raw images were captured for each sample and processed to ensure consistent white balance and exposure. The images were converted to JPEG format using the Adobe RGB 1998 color space, and the average RGB values were extracted from three different areas of each film using a Python script (Nizamov 2022). Considering the specific lighting conditions under which the images were captured, these values were also converted to the CIELAB color space (Nizamov 2022). This approach ensures a more perceptually uniform representation of color changes as perceived by human vision, whereas the RGB color space primarily represents the intensity of red, green, and blue light (Fairchild 2013). In CIELAB, L* represents lightness, ranging from black at 0 to white at 100. a* from green to magenta, and b* from blue to yellow. The ranges of a* and b* are theoretically unbounded but are often constrained in practical applications depending on the color gamut of the system used, as described in the sections that follow.

### Change in RGB

Color data for each sample were obtained from three distinct areas on both the initial and final images. These areas were specifically selected with the aim of representing different parts of the films, ensuring a comprehensive analysis of the color changes. The average pixel values for each area were calculated, and the color information was simplified into single RGB and subsequently into CIELAB data points. The RGB values from these areas were then averaged into a single initial RGB value and a single final RGB value. The color difference is quantified using the Euclidean distance—also referred to as ΔRGB—which is calculated using the formula

1 $$\Delta \text{RGB}=\sqrt{{({\text{R}}_{\text{Initial}}-{\text{R}}_{\text{Final}})}^{2}+{({\text{G}}_{\text{Initial}}-{\text{G}}_{\text{Final}})}^{2}+{({\text{B}}_{\text{Initial}}-{\text{B}}_{\text{Final}})}^{2}}$$

Equation (1) provides a numerical value that represents the overall change in color between the sample’s initial and final states as measured in the RGB color space. The standard deviation for ΔRGB is determined using the standard deviation of the color change vectors. This approach considers the variation in color changes across the three selected areas of the samples. A color change vector is computed for each area as the difference between the initial and final RGB values. The standard deviation (${\upsigma }_{\Delta RGB}$) of these three vectors provides a measure of how consistent or varied the color changes are across different areas of the film:

2 $${\sigma }_{\Delta RGB}=\sqrt{\frac{1}{n-1}{\sum }_{i=1}^{n}{\left(\Delta RG{B}_{i}-\overline{\Delta RGB}\right)}^{2}}$$

where ${\sigma }_{\Delta RGB}$ is the standard deviation of the color change vectors, $n$—is the number of areas, $\Delta RG{B}_{i}$—represents the Euclidean color difference for the i-th area, and $\overline{\Delta RGB}$—is the mean of the ΔRGB values across all areas.

### Perceived lightness change (L*)

The change in L* (ΔL*) in the CIELAB color space represents the change in the sample’s perceived lightness. The L* value quantifies a color’s lightness, ranging from 0 (complete black) to 100 (diffuse white). The formula used to calculate the change in lightness is as follows:

3 $$\Delta L*=L{*}_{\text{Initial}}-L{*}_{\text{Final}}$$

This value indicates how the sample’s perceived lightness has altered after exposure to accelerated aging tests light conditions. A positive ΔL* value indicates an increase in lightness, while a negative ΔL* value suggests a darkening of the sample. These ΔL* values’ standard deviation across the three areas is then calculated to quantify the uniformity of the lightness change. The formula for the standard deviation (${\upsigma }_{\Delta L*}$) is as follows:

4 $${\upsigma }_{\Delta L*}=\sqrt{\frac{1}{n-1}{\sum }_{i=1}^{n}{\left(\Delta L{*}_{i}-\overline{\Delta L*}\right)}^{2}}$$

where ${\upsigma }_{\Delta L*}$ is the standard deviation of the lightness change, $n$—is the number of areas, $\Delta L{*}_{i}$—represents the change in lightness for the i-th area, and $\overline{\Delta L*}$—is the mean change in lightness across all areas.

### Mechanical tensile, FTIR, and UV–vis measurements

Prior to mechanical testing, the films were preconditioned in a room for 72 h at 50% relative humidity and 25 °C. A Universal Tensile Tester (Instron 4204, Instron Corp., Norwood, MA, USA) was used to perform the mechanical tests. The samples were cut into rectangular shapes (15 mm × 5.3 mm) with 8 mm grips at both ends, and tests were performed with a 0.5 mm min^–1^ strain rate and repeated at least five times.

The FTIR measurements were performed using a PerkinElmer FTIR with ATR, and the UV–vis analysis was performed using the Diffuse Reflectance Accessory coupled with UV–Vis-NIR Agilent Cary 5000 from Agilent Technologies.

## Results and discussions

### Microscopy imaging

**Fig. 1:**
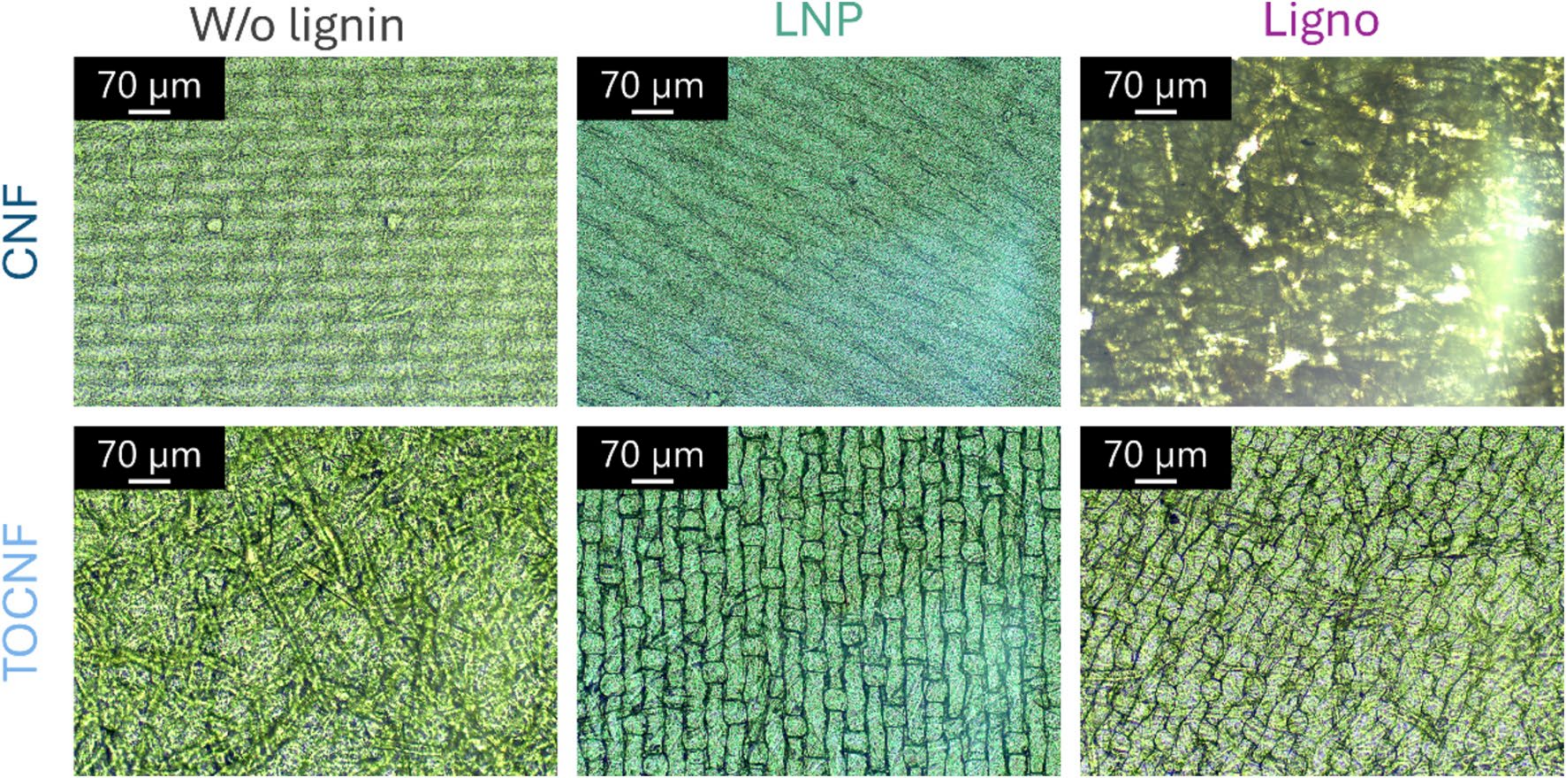
Optical micrographs of the NC-based films with and without lignin. The top row corresponds to CNF-based films, while the bottom row corresponds to TOCNF-based films

Large-scale morphological features of NC films taken using optical microscope are presented in Fig. 1. Although individual nanofibrils (tens of nanometers in diameter) cannot be resolved at this scale, the micrographs revealed that both CNF and TOCNF-based films formed dense, uniform fibrillar networks (Mattos et al. 2020). Furthermore, the shape of the filter used during the sample preparation can be seen from most of the samples. In lignin-containing films (CNF-LNP, TOCNF-LNP, LignoCNF, and LignoTOCNF), the optical micrographs revealed slightly more heterogeneous surface patterns compared to films without lignin (Fig. 1). These films often exhibited areas that appeared more uneven at the micron scale, potentially reflecting variations in local composition or fibrillar networks packing.

To further investigate the films’ morphology, FESEM was utilized (Figure S1). Despite the high resolution and the small field width (about 30 µm), distinct pores were not readily visible by FESEM, and this is likely due to the sputtered coating needed to make the film conductive for the imaging. Consequently, density was measured based on weight and dimensions to give further information about the internal structure and from there even the porosity can be estimated (Table S1).

### Visual and quantitative color changes

**Fig. 2:**
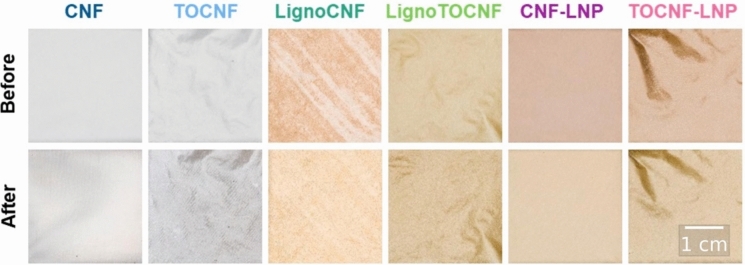
Digital photographs of nanocellulose films before and after 1000 h exposure to light (Xenon light—corresponding to AM1.5G spectrum)

**Fig. 3:**
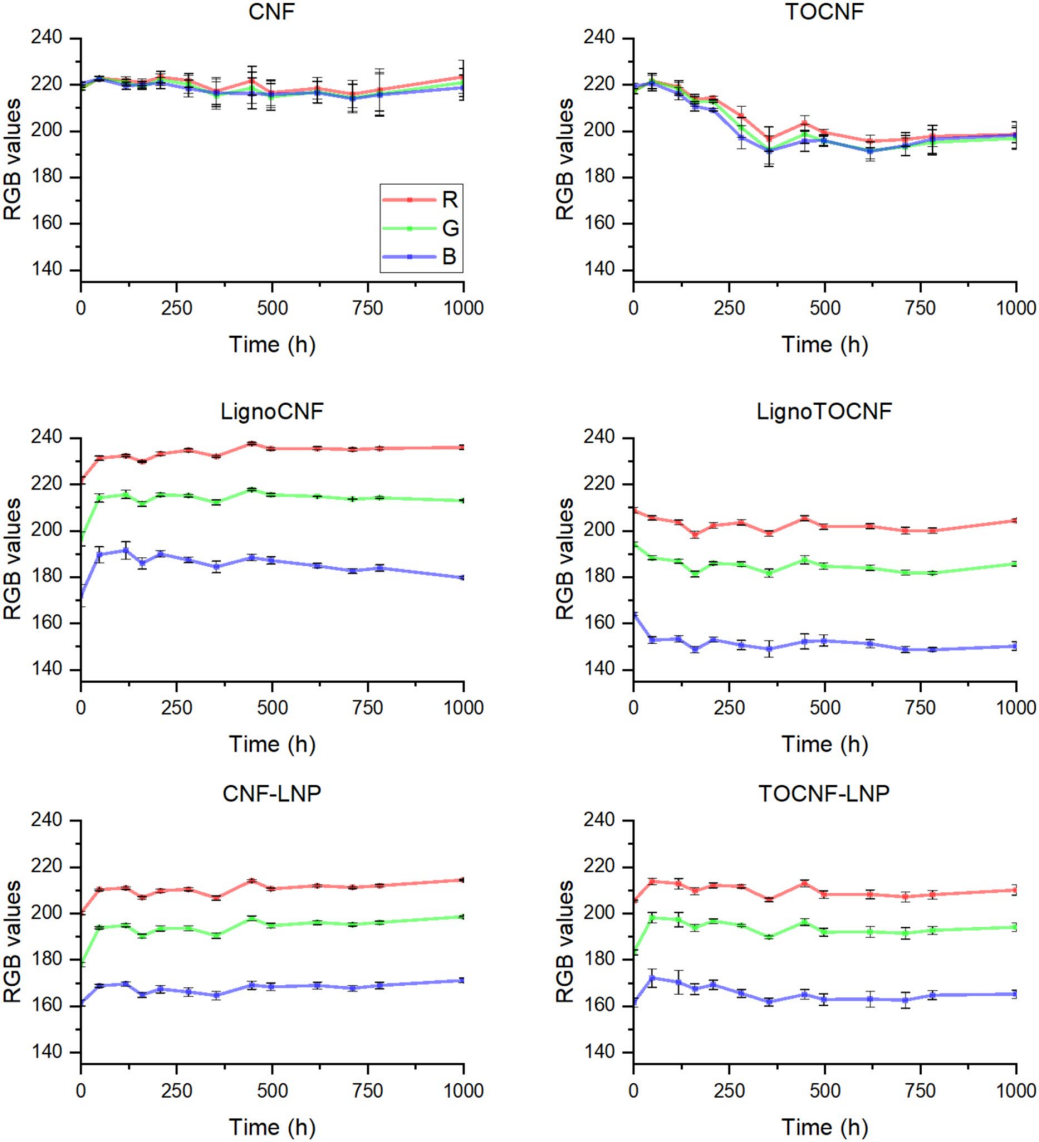
The variation of red, green, and blue (RGB) values over time for nanocellulose films with standard deviation. The plots indicate the changes in RGB intensity levels during a 1000-h accelerated aging test, with error bars representing the standard deviation for three distinct regions of each film, ensuring data uniformity. The y-axes of the plots are truncated to display a range from 135 to 240, enhancing the visibility of trends in the data

As Fig. 2 illustrates, CNF and TOCNF exhibited the anticipated features of a translucent nanopaper with white nuances, and LignoCNF, LignoTOCNF, CNF-LNP, and TOCNF-LNP films exhibited the distinctive brownish color associated with lignin-based materials. Overall, the visual appearance of all NC films remained largely unchanged after having been exposed to light exposure. As Fig. 3 illustrates, most of the color changes occurred between the first two data points (approximately first 50 h).

Quantitatively, the Euclidian distance between initial and post-exposure RGB values and the difference between initial and after 1000 h of exposure, L* values of CIELAB are congruent with the visual appearance of the films (Table 1). The standard deviations in ΔRGB and ΔL* highlight natural surface heterogeneity and variations in local material properties across three distinct areas of each film (refer Figure S2). ΔRGB represents the overall vectorial shift in color, capturing changes in hue and saturation, while its standard deviation reflects the variability in color changes across regions. In case of the CNF film, a high standard deviation relative to the ΔRGB value (Table 1) suggests minimal overall color shift but reduced surface homogeneity. In contrast, ΔL* measures only the scalar lightness component, independent of hue and saturation. Lower ΔL* standard deviations suggest more uniform lightness changes.

**Table 1:** Quantitative analysis of color alterations in the NC films, showing both ΔRGB (change in color composition in RGB color space) and ΔL* (change in perceived lightness in CIELAB color space) after 1000 h of exposure to artificial sunlight

	CNF	TOCNF	LignoCNF	LignoTOCNF	CNF-LNP	TOCNF-LNP
ΔRGB	6 ± 6	35 ± 7	23 ± 5	17 ± 4	26.9 ± 0.8	13 ± 4
ΔL*	0.6 ± 1.1	− 3.5 ± 0.8	2.7 ± 0.5	− 1.3 ± 0.2	3.3 ± 0.2	1.6 ± 0.4

The aged TOCNF showed noticeable darkening, which was also evidenced by a decrease in the RGB values (Fig. 3) and negative ΔL* (Figure S3). Notwithstanding minor variations in hue and saturation, the films containing lignin exhibited distinct changes in color lightness upon exposure to light: LignoCNF, CNF-LNP, and TOCNF-LNP became lighter, while the LignoTOCNF film darkened. The lightening of the lignin-containing films can be explained by the photobleaching of lignin chromophores under UV exposure. During this process, phenolic groups in lignin are converted into phenoxyl radicals, which may further react to form quinones. Although quinones contribute to color, extended UV exposure can degrade them into less colored aliphatic acid structures, resulting in increased lightness (positive ΔL*) (Barclay et al. 1998). Conversely, the darkening observed in the LignoTOCNF film (negative ΔL*) can be associated with the formation of new chromophoric structures upon UV irradiation. This aligns with the photo-yellowing mechanism, where lignin undergoes oxidative reactions involving singlet oxygen, forming additional chromophores like quinones that darken the material (Xing et al. 2017).

### Optical properties: transmittance and haze

**Fig. 4:**
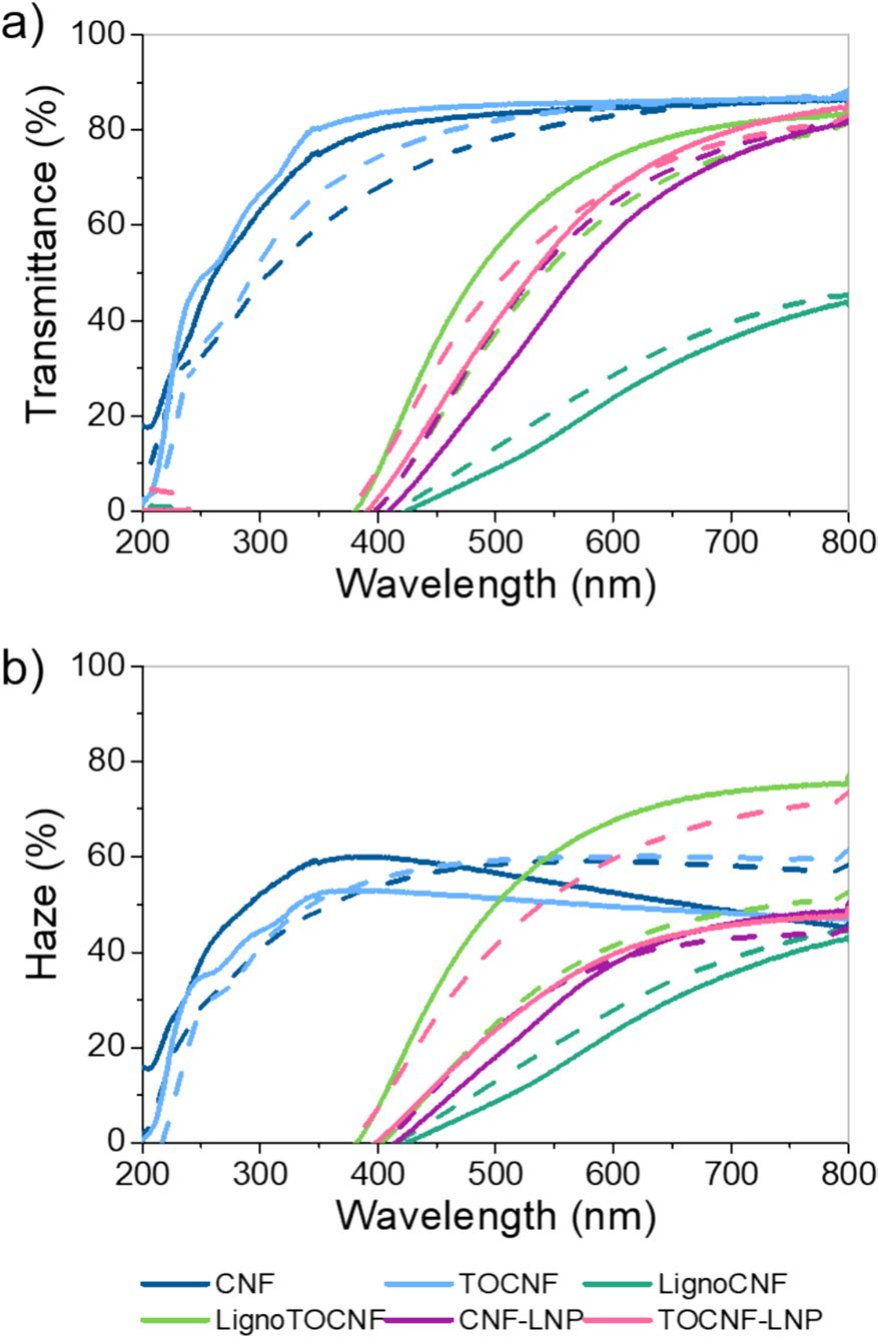
**a** Transmittance and **b** haze before (solid line) and after (dashed line)1000 h of exposure to artificial sunlight

Color changes seen in the photos may be associated with alterations in the optical properties of the films. To explore these changes, we measured transmittance and haze (see Fig. 4). As expected, films without lignin (CNF and TOCNF) exhibited the highest transmittance (around 80% visible range) with a UV absorbance that became significant below 300 nm (Fig. 4-a). Despite their apparent color stability (Fig. 2), both films experienced a reduction in transmittance after aging, particularly below 600 nm.

Despite differences in thickness (Table S2) and the lignin’s presentation (i.e., residual or nanoparticles), all films containing lignin exhibited outstanding UV-blocking properties extending up to 400 nm. However, these films exhibited subpar performance within the visible spectrum, meaning that they were less transparent than CNF and TOCNF. The film with the lowest transparency was LignoCNF, showing 15% transmittance at 550 nm, followed by CNF-LNP with 44% transmittance. Following their exposure to light, among all the films that contained lignin, LignoTOCNF alone showed a decrease in transmittance, which may be related to the color changes observed above. In some applications, gaining UV-blocking properties with biodegradable materials may be worth sacrificing a part of visible light transmittance.

All NC films exhibited light-scattering features indicating optical haze (Fig. 4-b). Haze essentially measures the amount of light that is scattered as a beam of light that travels through a material (film). This property varied significantly among the films owing to its heavy dependence on the structure, fibril size distribution, porosity, and the presence of additives (Jacucci et al. 2020). Upon light exposure, notable alterations in haze were observed; CNF and TOCNF films experienced a considerable increase in haze for wavelengths above 450 nm. Additionally, TOCNF-LNP exhibited a significant increase in haze while LignoTOCNF showed a reduction of approximately 30%. Both CNF-LNP and LignoCNF presented minimal changes compared with the films mentioned above.

These variations are likely the result of structural alterations caused by the exposure to light during the experiment. Although the sample temperature remains well below the boiling point of water (45 °C), continuous exposure to sunlight can gradually evaporate moisture within the film’s structure. This moisture loss causes structural modifications in the material that may be responsible for the alterations in the haze of these materials (Qing et al. 2013; Isobe et al. 2018; Li et al. 2020).

### UV protection efficacy

**Fig. 5:**
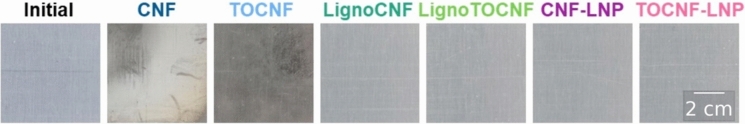
Digital imaging of the polyvinyl chloride plastic used behind the films following 1000 h of exposure in comparison to the initial one

**Fig. 6:**
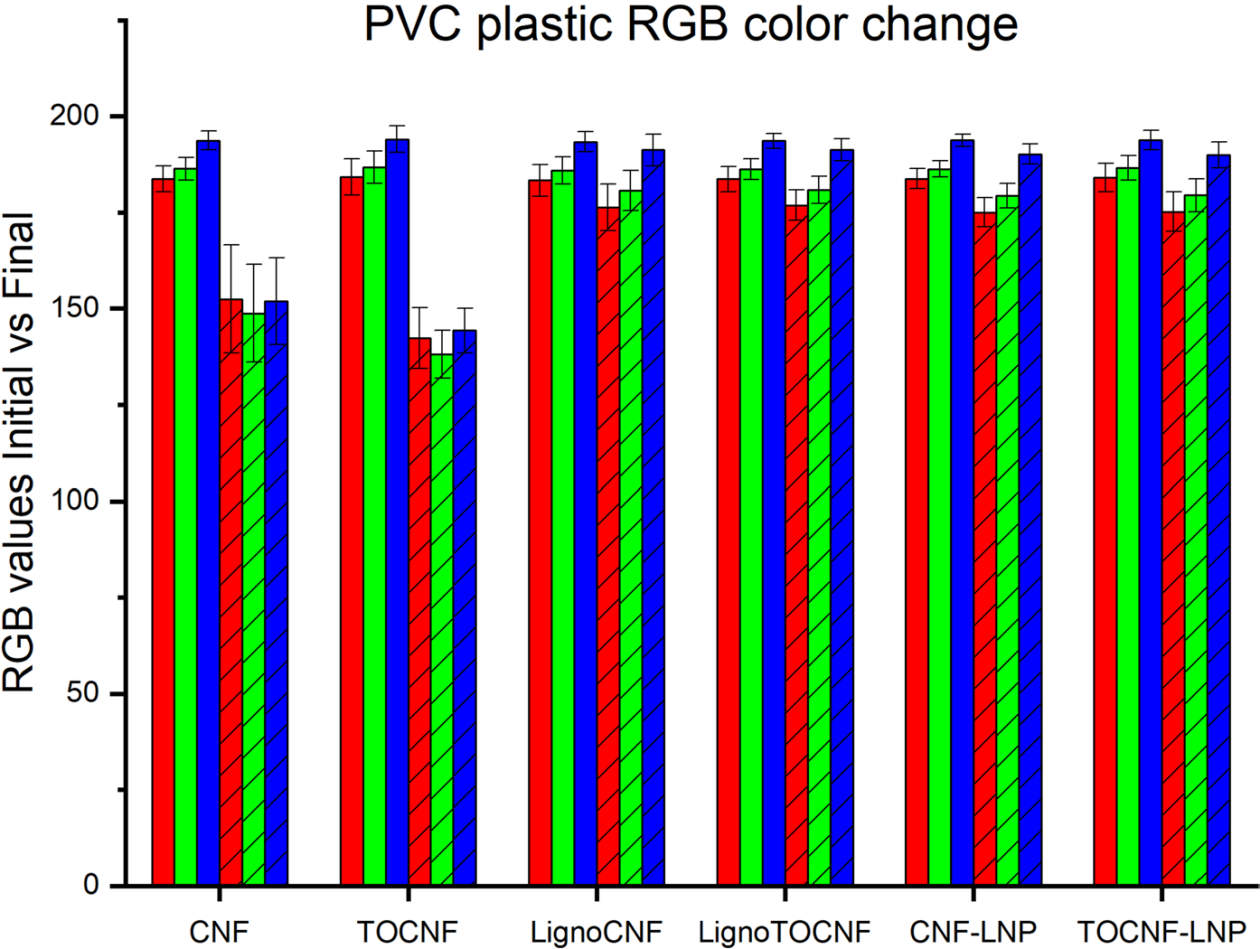
Changes in RGB color values for PVC plastic samples covered by various nanocellulose (NC) films before and after 1000 h of exposure to artificial sunlight. The bar graph compares the initial (solid bars) and final (hatched bars) RGB values for each nanocellulose (NC) film: CNF, TOCNF, LignoCNF, LignoTOCNF, CNF-LNP, and TOCNF-LNP. The RGB color values (red, green, and blue) are presented for each sample, with error bars indicating the standard deviation across three distinct regions

In the context of optoelectronic devices, NC films that are enhanced with lignin molecules offer not only improved light management through transmittance and haze but also crucial UV protection. To assess this UV shielding’s effectiveness, we placed NC films on top of rigid polyvinyl chloride (PVC) samples and exposed them to light for 1000 h and subsequently evaluated the color conversion of the PVC (Fig. 5). PVC is known to suffer from physical and chemical changes by long exposure to UV irradiation, inducing color alteration (Hadi et al. 2019). As anticipated, both CNF and—particularly—TOCNF films are poor at shielding UV light, which is known to cause photo-oxidative aging and significant deterioration of synthetic polymers (Lu et al. 2018). By contrast, PVC samples covered with lignin-containing films exhibited less color alternation (Fig. 6, Table S3, Figure S4). When this understanding is extrapolated to the realm of optoelectronics, empirical evidence indicates that NC films containing lignin offer substantial and enduring UV-blocking attributes that can improve such devices’ lifetime performance.

### Mechanical properties alternations

In flexible optoelectronics, even slight alterations in a device’s mechanical properties can significantly impact its production process, and during use, the mechanical requirements vary depending on the application. Surprisingly, the alterations in the mechanical properties caused by extensive exposure to light have been largely overlooked in research. Existing research has documented chemical modifications and reductions in polymerization levels in materials such as wood (Cogulet et al. 2016), cellulose, and lignocellulosic materials (Liu et al. 2019). Both types of modifications could induce alteration of their mechanical properties which have been neglected. The present study, therefore, also assessed the NC films’ mechanical properties before and after light exposure.

**Fig. 7:**
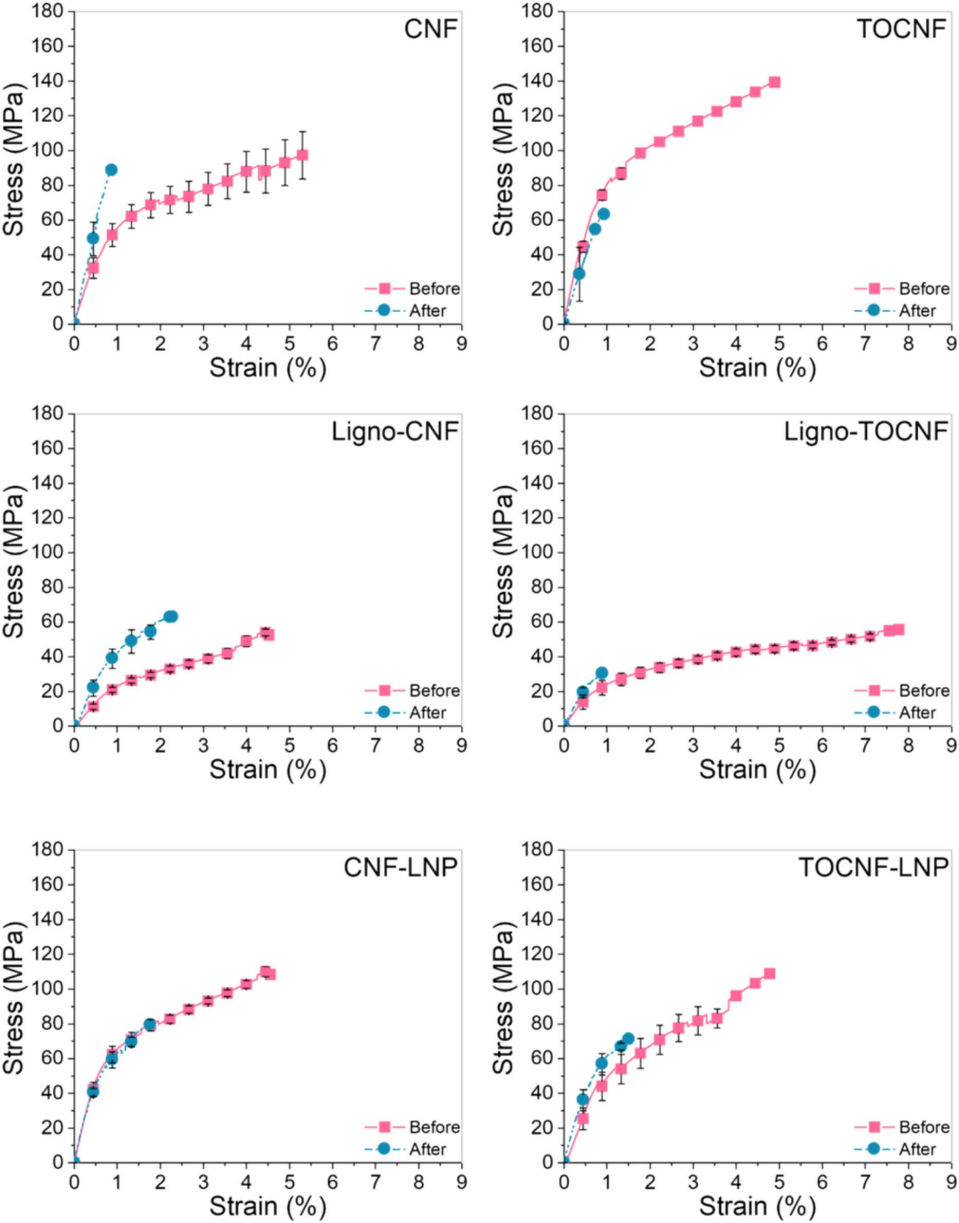
Mechanical properties of NC cellulose before and after light exposure. All measurements were performed with the same conditions (50% relative humidity, 25 °C and 0.5 mm min^–1^ strain rate) and repeated at least five replicates

As Fig. 7illustrates, all NC films underwent significant changes in their tensile strain as a result of their exposure to artificial sunlight, regardless of the lignin type or presence in their structure. Qualitatively CNF films containing lignin were least affected by the sunlight exposure, indicating a greater stability.

All the films became stiffer than confirmed by Young Modulus values (Table S2), although the underlying factors contributing to this change remain unclear. For instance, the Young Modulus values of TOCNF and CNF-LNP increased less, indicating that both films became less brittle than the other films. This could indicate differences in fiber interactions and structural formations within the film. Following this assumption, in agreement with previous work (Kaschuk et al. 2024), TOCNF is highly fibrillated and can produce material with higher brittleness compared to CNF films in consequence of the fibril size and presence of carboxyl groups. The entanglement and surface interactions among the fibrils may be a reason for the increased stiffness of TOCNF film before and after light exposure. Additionally, Pasquier et. al. (Pasquier et al. 2021) studied how the surface interactions between CNF and the lignin nanoparticles creates a “phase separation” within the structure of the film during the nucleation of the LNP which could be reason the CNF-LNP presented the lowest change on stiffness. Furthermore, it was observed that in both Ligno-CNF and Ligno-TOCNF films which contain native lignin became stiffer after the exposure than the other films.

There are several factors which could induce the changes observed in the mechanical properties of the NC films. For instance, both cellulose and lignin suffer oxidation under light exposure (Cogulet et al. 2016; Qi et al. 2016) leading to decrease of molar mass and in consequence mechanical alterations. Also, exposure to light causes modified cellulose to generate more free radicals, accelerating oxidation and degradation, which leads to greater changes in mechanical properties (Daruwalla et al. 1972).

Here, we used the FTIR to recognize potential photodegradation after light exposure. All CNF films exhibited a peak intensity in infrared spectra between 1700 and 1750 cm^−1^(Figure S5), which may be attributed to the C = O stretching vibration of aldehyde groups (Singh et al. 2023) caused by the oxidation of cellulose (Łojewska et al. 2005). Additionally, after exposure, TOCNF films presented higher intensity on characteristic cellulose peaks for hydrogen-bonded O–H stretching (~ 3300 cm^−1^) and for sp3 hybridized C–H stretching (~ 2900 cm^−1^) indicating a decrease of the carboxyl content on the NC films.

## Conclusion

This study provides insights into the color, optics, and mechanical properties of NC films with and without lignin following prolonged exposure to light. Most significantly, only minimal changes were observed in the colors and optical properties of NC films after exposure to sunlight. Most of these minor changes occurred very early (within approximately 50 h) during the light exposure and remained unaltered afterward. This good stability indicates that NC films are promising alternatives as substrates for use in optoelectronics, such as solar cells. These films offer enduring optical performance, particularly when lignin is used as a UV-blocking particle. Lignin demonstrated stability in UV-blocking, regardless of its form within the film. Consequently, this finding demonstrated that even after it has been removed from nature, lignin continues to provide long-lasting UV protection.

Nevertheless, all films, regardless of the presence of lignin, exhibited modifications in their mechanical properties following light exposure. Some films became marginally more brittle, while all films exhibited a decrease in strain at break, likely due to the oxidation of both cellulose and lignin components. It is worth noting that mechanical properties are particularly important in the manufacturing of solar devices (i.e., before aging), but its significance in practice varies considerably according to the application and may be only minor. Overall, the incorporation of lignin into NC films activates their UV-blocking capabilities and significantly enhances their mechanical stability, highlighting their potential for use in sustainable optoelectronic applications.

## Supplementary Information

Below is the link to the electronic supplementary material.

Supplementary file1 (DOCX 1319 KB)

## Data Availability

No datasets were generated or analysed during the current study.

## Abbreviations

UV: Ultraviolet
NC: Nanocellulose
OLED: Organic light-emitting diode
CNF: Cellulose nanofibrils
TOCNF: Tempo-oxidized cellulose nanofibrils
CNF-LNP: Cellulose nanofibrils with lignin nanoparticles
TOCNF-LNP: Tempo-oxidized cellulose nanofibrils with lignin nanoparticles
LignoCNF: Cellulose nanofibrils with residual lignin
LignoTOCNF: Tempo-oxidized cellulose nanofibrils with residual lignin

## Declarations

### Associated content

Supporting Information. This material is available free of charge. Figure S1: FESEM images of the NC films. Table S1: Density and porosity analysis of the NC films. Figure S2: Representative example of a digital photograph showcasing the selected areas (black rectangles) for the LignoTOCNF film. Figure S3: Variation of CIELAB values over time for nanocellulose films with standard deviation; Table S2: Thickness of the films; Table S3: ΔRGB and *ΔL** of in the PVC samples; Figure S4: Changes in CIELAB color values for PVC plastic samples; Figure S5: FTIR spectra of nanocelluloses films before and after light exposure.
