# Supplementary material for: Optical assessment of lignin-containing nanocellulose films under extended sunlight exposure
Source: Cellulose (Lond). 2025 Jan 15;32(9):5321–34. doi: 10.1007/s10570-025-06380-7 (PMC12254080; doi:10.1007/s10570-025-06380-7)
Supplement: Supplementary file 1 — Supplementary file1 (DOCX 1319 KB) [file 10570_2025_6380_MOESM1_ESM.docx]

**Supporting Information**

Optical assessment of lignin-containing nanocellulose films under extended sunlight exposure

Rustem Nizamov^1#^, Joice Kaschuk^2,3,4#*^, Yazan Al Haj^5^, Mikael Nyberg^1^, Monireh Imani^3,6^, Eva Pasquier^3^, Orlando Rojas^3,4^, Tiffany Abitbol^7^, Jaana Vapaavuori^5^, Kati Miettunen^1^

1. Department of Mechanical and Materials Engineering, Faculty of Technology, University of Turku, Turku, FI-20500 Finland

2. Physical Chemistry and Soft Matter, Wageningen University and Research, 6708 WE, Wageningen, The Netherlands.

3. Department of Bioproducts and Biosystems, School of Chemical Engineering, Aalto University, Vuorimiehentie 1, 02150 Espoo, Finland

4. Department of Chemical and Biological Engineering, The University of British Columbia, 2360 E Mall, V6T 1Z3, Vancouver - BC, Canada.

5. Department of Chemistry and Materials Science, School of Chemical Engineering, Aalto University, Kemistintie 1, 02150 Espoo, Finland.

6. Mirka Ltd., Pensalavägen 210, FI-66850 Jeppo, Finland

7. Institute of Materials, School of Engineering, École Polytechnique Fédérale de Lausanne (EPFL), 1015, Switzerland.

# contributed equally to this work.

* Correspondent author: [joice.kaschuk@wur.nl](mailto:joice.kaschuk@wur.nl)

KEYWORDS: stability, optoelectronics, mechanical properties, UV protection, biobased solar cells.

**
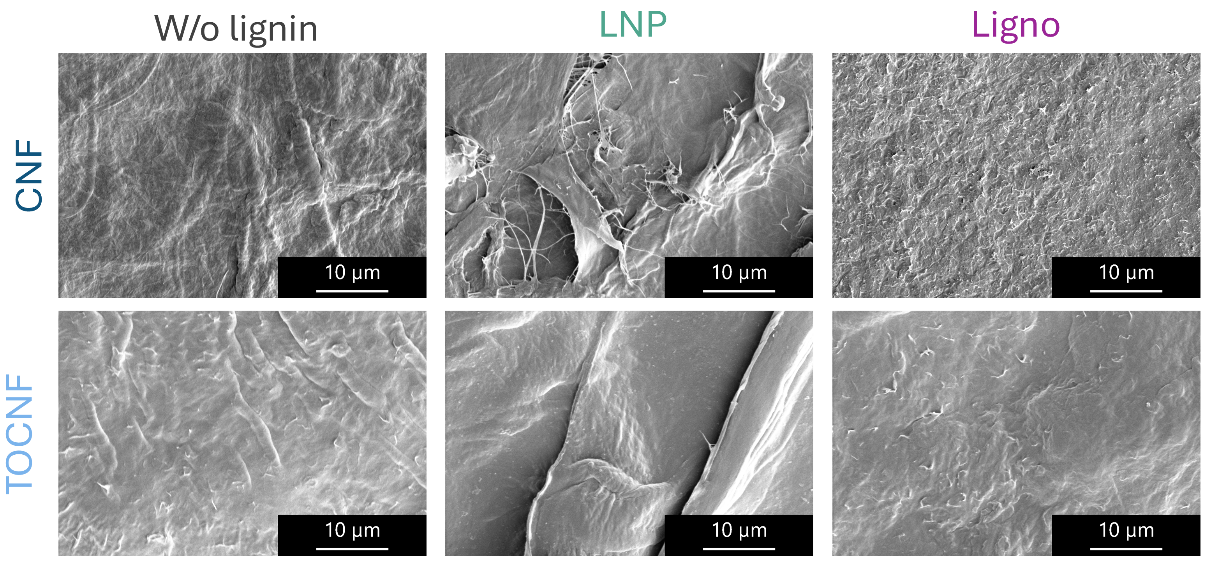
**

**Figure S 1** Surface morphologies of the NC-based films with and without lignin. The top row corresponds to CNF-based films, while the bottom row corresponds to TOCNF-based films.

Table S 1 Density and porosity analysis of the NC films

| Sample | Mass (mg) | Area (mm^2^) | Volume (mm^3^) | Density (g/cm^3^) |
| --- | --- | --- | --- | --- |
| CNF | 4.9 | 113 | 4.52 | 1.09 |
| LignoCNF | 6.6 | 84 | 5.04 | 1.32 |
| CNF-LNP | 5.6 | 99 | 4.95 | 1.13 |
| TOCNF | 5.9 | 109 | 4.36 | 1.35 |
| LignoTOCNF | 5.3 | 114 | 9.12 | 0.58 |
| TOCNF-LNP | 3.9 | 73 | 3.65 | 1.06 |

The samples were cut into rectangular pieces of approximately 1 cm² in size. Their mass was measured using a high-precision laboratory analytical scale (Sartorius BCE224I-1S). To determine their areas, digital images of the samples were analyzed using ImageJ software version 1.54g (National Institutes of Health, USA), with appropriate calibration to ensure measurement accuracy. Using the thickness values provided in Table 2, the volumes of the samples were calculated based on the formula:

$$\text{Volume} \left( \text{cm}^{3} \right)=\text{Area} \left( \text{cm}^{2} \right)\times\text{Thickness} \left( \text{cm} \right)$$

Subsequently, the density (ρ) of each film was calculated as:

$$\rho\left( {\text{g}\text{/}\text{cm}}^{3} \right)=\frac{\text{Mass} \left( \text{g} \right)}{\text{Volume} \left( \text{cm}^{3} \right)}$$

If we assume porosity of cellulose fibers as 1.61 g/cm^3^ (Antlauf et al., 2021), the estimate porosity for the samples will vary between 15-60%.


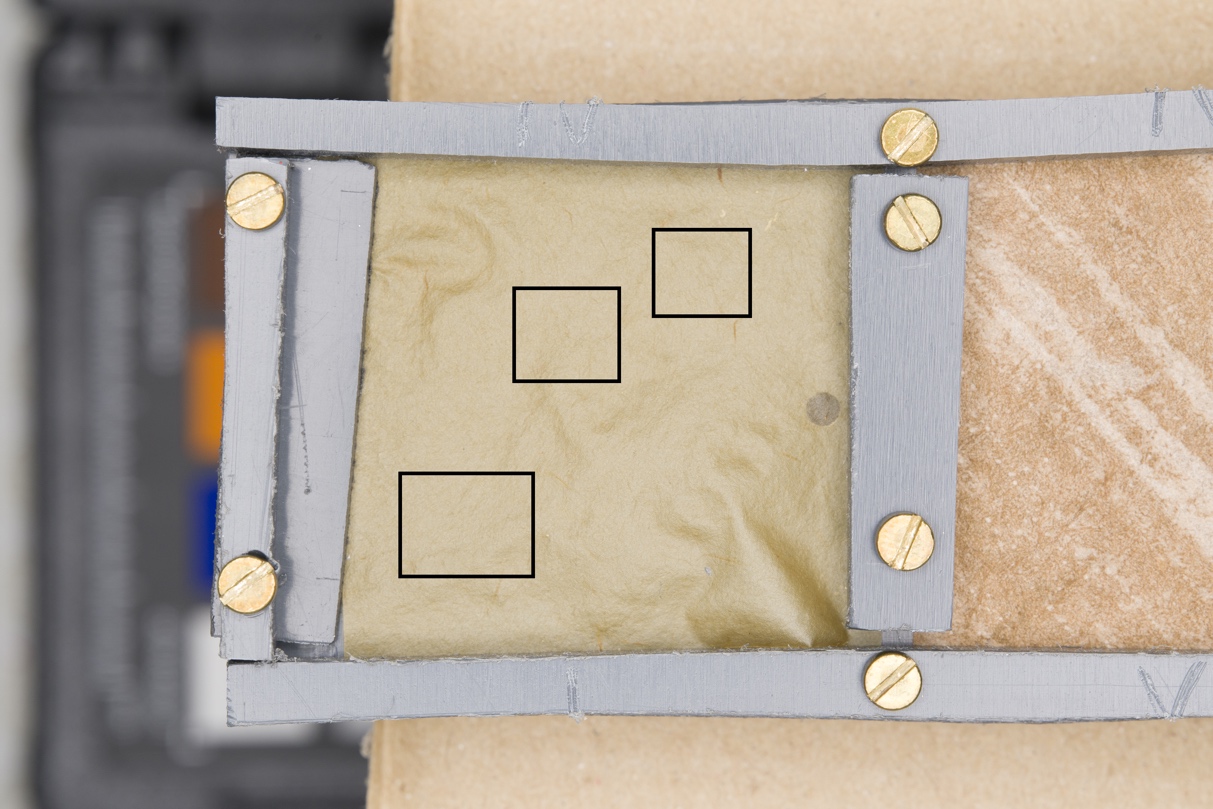
 **Figure S 2 -** Representative example of a digital photograph showcasing the selected areas (black rectangles) for the LignoTOCNF film.


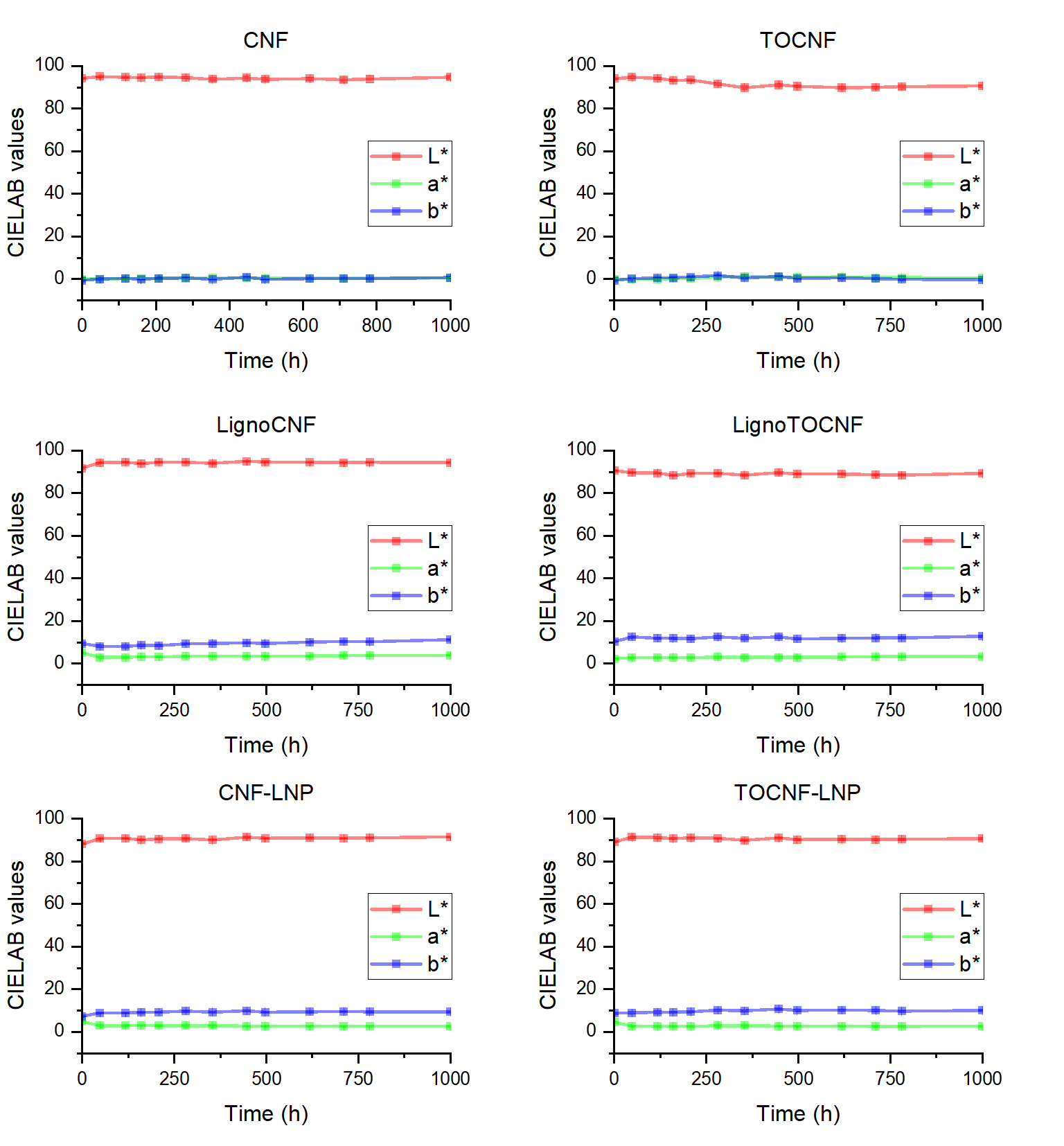


**Figure S 3 -** Variation of CIELAB values over time for nanocellulose films with standard deviation. The plots show the changes in lightness (L*), green-red component (a*), and blue-yellow component (b*) during a 1000-hour accelerated aging test. The L* value represents lightness ranging from black (0) to white (100), a* indicates the green to red spectrum, and b* indicates the blue to yellow spectrum. Error bars represent the standard deviation for three distinct regions of each film, ensuring uniformity in the data.

Table S 2 – Thicknesses and Young Modulus of the NC films. The uncertainty in thickness values represents the standard deviation derived from multiple measurements. Similarly, Young Modulus uncertainties are standard deviations calculated from 5 replicate tests.

| Sample | Thickness (mm) | Young Modulus (MPa) before | Young Modulus (MPa) after | Increase of YM (%) |
| --- | --- | --- | --- | --- |
| CNF | 40 ± 1 | 8600 ± 1600 | 13000 ± 700 | 35 |
| LignoCNF | 60 ± 2 | 3300 ± 400 | 6000 ± 400 | 44 |
| CNF-LNP | 50 ± 1 | 10900 ± 1600 | 11000 ± 700 | 1.5 |
| TOCNF | 40 ± 2 | 9200 ± 1400 | 9400 ± 600 | 2.7 |
| LignoTOCNF | 80 ± 5 | 3200 ± 900 | 4600 ± 1000 | 29 |
| TOCNF-LNP | 50 ± 1 | 7500 ± 1500 | 8500 ± 2000 | 11 |

Table S 2 - Quantitative analysis of color alterations in the PVC samples covered by NC films, showing both ΔRGB and *ΔL** after 1000 h of exposure to artificial sunlight. The uncertainty represents the standard deviation between three distinct areas of the films.

|  | CNF | TOCNF | LignoCNF | LignoTOCNF | CNF-LNP | TOCNF-LNP |
| --- | --- | --- | --- | --- | --- | --- |
| ΔRGB | 64 ± 24 | 81 ± 7 | 9 ± 8 | 9 ± 5 | 12 ± 7 | 12 ± 8 |
| *ΔL** | -7 ± 2.9 | -9.5 ± 1.8 | -1.1 ± 1.2 | -1.1 ± 0.9 | -1.4 ± 0.8 | -1.4 ± 1.1 |


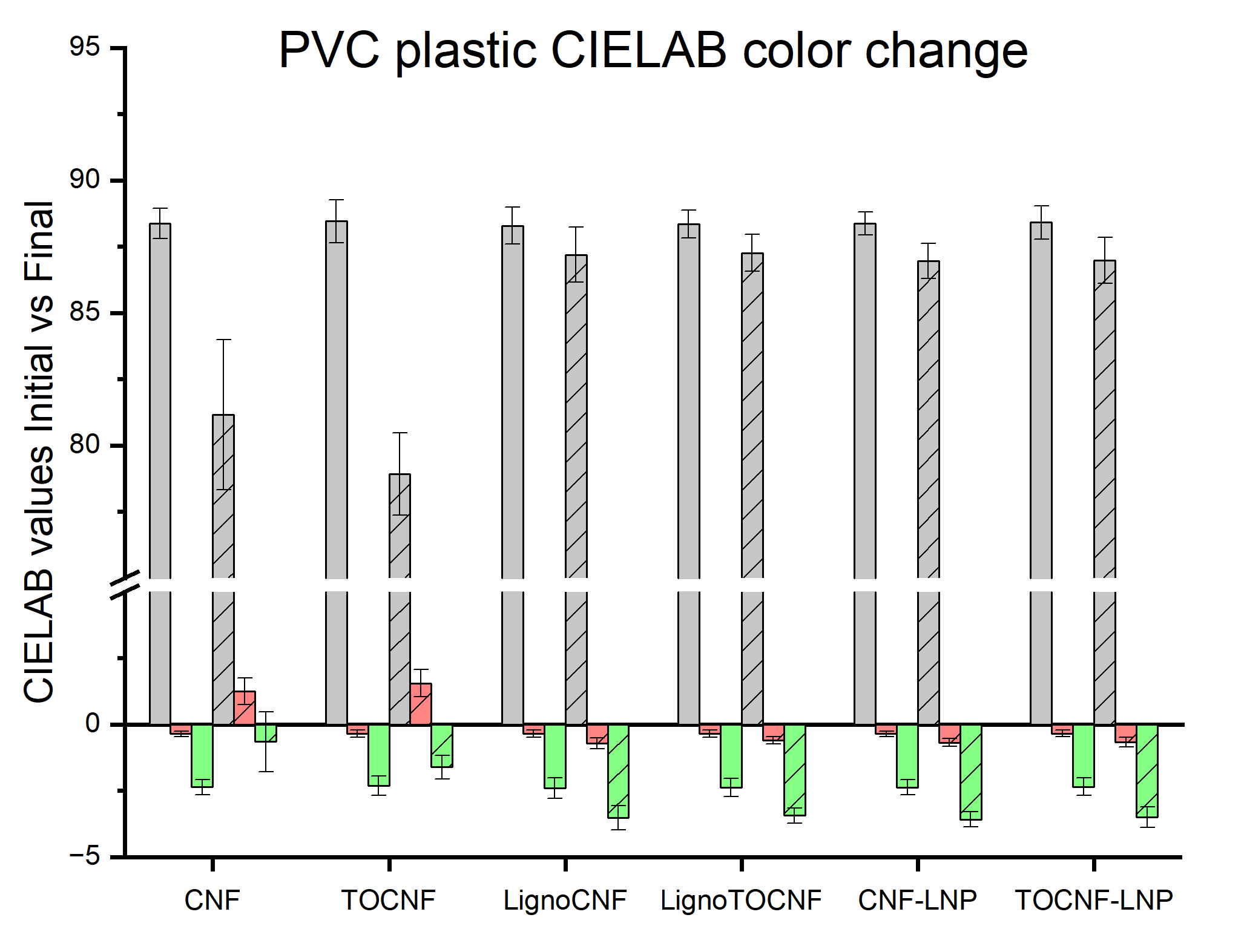


**Figure S 4 -** Changes in CIELAB color values for PVC plastic samples covered by various nanocellulose (NC) films before and after 1000 hours of exposure to artificial sunlight. The data is represented for different NC films: CNF, TOCNF, LignoCNF, LignoTOCNF, CNF-LNP, and TOCNF-LNP. The bar graph shows the CIELAB values for L* (gray), a* (red), and b* (green) for each sample. The initial values are solid bars, while the final values after exposure are hatched bars. Error bars represent the standard deviation across three distinct regions of each sample, providing insight into the uniformity of color changes.


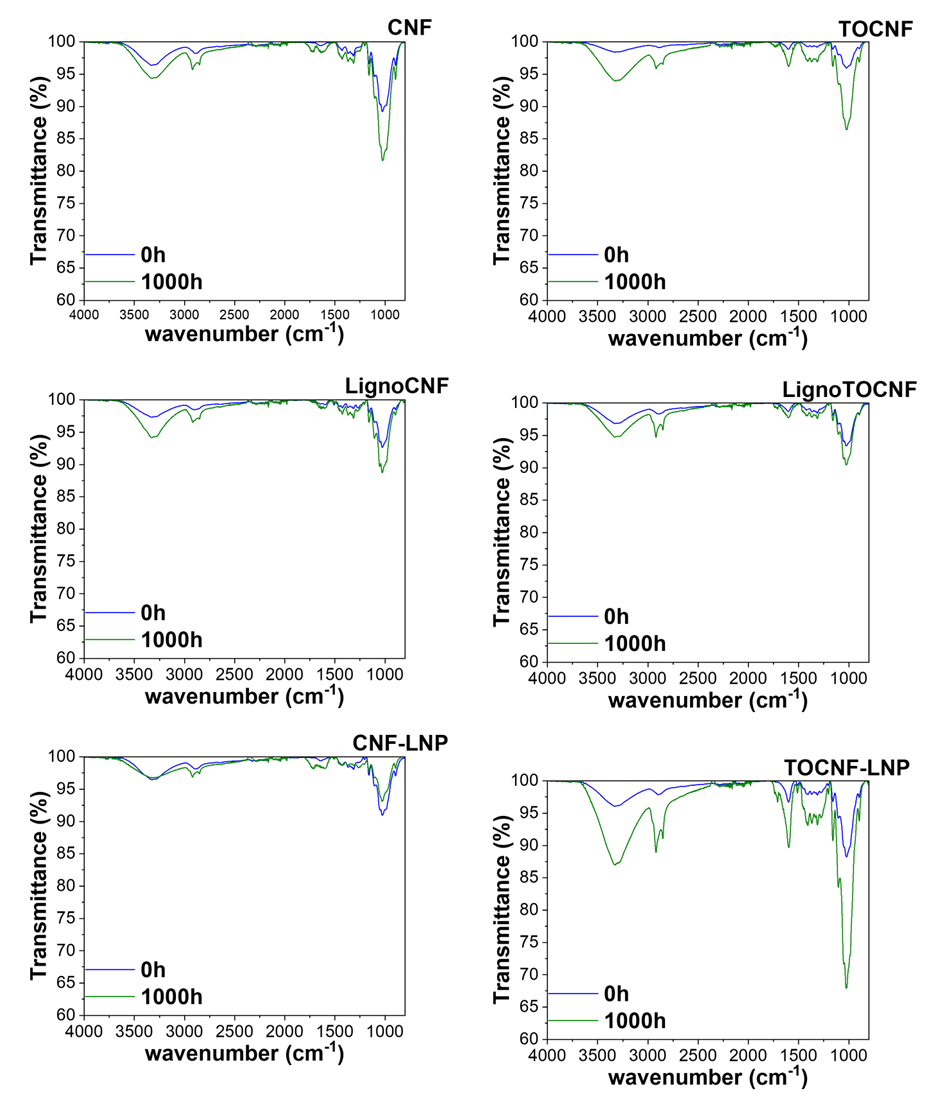


**Figure S 5 -** FTIR spectra of nanocellulose films before and after light exposure

REFERENCES

Antlauf, M., Boulanger, N., Berglund, L., Oksman, K., & Andersson, O. (2021). Thermal Conductivity of Cellulose Fibers in Different Size Scales and Densities. *Biomacromolecules*, *22*(9). https://doi.org/10.1021/acs.biomac.1c00643
